# Association Between Plasma Apolipoprotein M With Alzheimer’s Disease: A Cross-Sectional Pilot Study From China

**DOI:** 10.3389/fnagi.2022.838223

**Published:** 2022-03-18

**Authors:** Jia-Yan Xin, Xiao Huang, Ying Sun, Hai-Song Jiang, Jin Fan, Neng-wei Yu, Fu-Qiang Guo, Fang Ye, Jun Xiao, Wei-dong Le, Shao-Jie Yang, Yang Xiang

**Affiliations:** ^1^Department of Clinical Medicine, North Sichuan Medical College, Nanchong, China; ^2^Department of Neurology, General Hospital of Western Theater Command, Chengdu, China; ^3^Department of Geriatrics, Sichuan Provincial People’s Hospital, University of Electronic Science and Technology of China, Chengdu, China; ^4^Chinese Academy of Sciences Sichuan Translational Medicine Research Hospital, Chengdu, China; ^5^Department of Neurology, Sichuan Provincial People’s Hospital, University of Electronic Science and Technology of China, Chengdu, China; ^6^Department of Neurology, Chengdu Eighth People’s Hospital, Chengdu, China

**Keywords:** Alzheimer’s disease, apolipoprotein M, neurofilament light chain, tau, biomarker

## Abstract

**Background:**

Recent evidence of genetics and metabonomics indicated a potential role of apolipoprotein M (ApoM) in the pathogenesis of Alzheimer’s disease (AD). Here, we aimed to investigate the association between plasma ApoM with AD.

**Methods:**

A multicenter, cross-sectional study recruited patients with AD (*n* = 67), age- and sex-matched cognitively normal (CN) controls (*n* = 73). After the data collection of demographic characteristics, lifestyle risk factors, and medical history, we examined and compared the plasma levels of ApoM, tau phosphorylated at threonine 217 (p-tau217) and neurofilament light (NfL). Multivariate logistic regression analysis was applied to determine the association of plasma ApoM with the presence of AD. The correlation analysis was used to explore the correlations between plasma ApoM with cognitive function [Mini–Mental State Examination (MMSE) and Montreal Cognitive Assessment (MoCA)], activities of daily living (ADL), and the representative blood-based biomarkers (plasma p-tau217 and NfL). Receiver operating characteristic (ROC) analysis and Delong’s test were used to determine the diagnostic power of plasma ApoM.

**Results:**

Plasma ApoM and its derived indicators (ratios of ApoM/TC, ApoM/TG, ApoM/HDL-C, and ApoM/LDL-C) were significantly higher in AD group than those in CN group (each *p* < 0.0001). After adjusted for the risk factors of AD, the plasma ApoM and its derived indicators were significantly associated with the presence of AD, respectively. ApoM (OR = 1.058, 95% CI: 1.027–1.090, *p* < 0.0001), ApoM/TC ratio (OR = 1.239, 95% CI: 1.120–1.372, *p* < 0.0001), ApoM/TG ratio (OR = 1.064, 95% CI: 1.035–1.095, *p* < 0.0001), ApoM/HDL-C ratio (OR = 1.069, 95% CI: 1.037–1.102, *p* < 0.0001), and ApoM/LDL-C ratio (OR = 1.064, 95% CI:1.023–1.106, *p* = 0.002). In total participants, plasma ApoM was significantly positively correlated with plasma p-tau217, plasma NfL, and ADL (each *p* < 0.0001) and significantly negatively correlated with MMSE and MoCA (each *p* < 0.0001), respectively. In further subgroup analyses, these associations remained in different *APOE*ϵ 4 status participants and sex subgroups. ApoM/TC ratio (ΔAUC = 0.056, *p* = 0.044) and ApoM/TG ratio (ΔAUC = 0.097, *p* = 0.011) had a statistically remarkably larger AUC than ApoM, respectively. The independent addition of ApoM and its derived indicators to the basic model [combining age, sex, *APOE*ϵ 4, and body mass index (BMI)] led to the significant improvement in diagnostic power, respectively (each *p* < 0.05).

**Conclusion:**

All the findings preliminarily uncovered the association between plasma ApoM and AD and provided more evidence of the potential of ApoM as a candidate biomarker of AD.

## Introduction

As characterized by the progressive decline of cognitive function, activities of daily living (ADL), and social function (Knopman et al., 2021), Alzheimer’s disease (AD) is an age-related neurodegenerative disorder, which accounts for about 60–80% of all dementias (Alzheimer’s Association, 2021). It is generally believed that the sporadic AD (which accounts for more than 99% of all cases) is comprehensively caused by age, genetics, metabolism, inflammation, and environmental and other factors (Livingston et al., 2020); however, the exact pathogenesis has not been fully elucidated and the treatment that can block or reverse the disease progress has not been developed so far (Levey, 2021). Thus, the significance of the early diagnosis of AD grows to be increasingly prominent.

In the past decades, the great progress of biomarkers has promoted an early diagnosis of AD to a large extent. The AD research framework proposed by NIA-AA in 2018 categorized AD biomarkers into A/T(N) system (Jack et al., 2018), in which the core biomarkers were mainly based on cerebrospinal fluid (CSF) test [e.g., CSF-amyloid β (Aβ) and CSF-phosphorylated-tau (p-tau)] or positron emission tomography (PET) test (e.g., ^11^C-Pittsburgh compound B (PiB)-PET and tau-PET) (Jack et al., 2018). Nevertheless, the potential risk of invasive deep puncture in CSF collection and the high cost of PET test have hindered the applications of these biomarkers. Therefore, the development of blood-based biomarkers of AD is promising currently (Thijssen and Rabinovici, 2021).

Apolipoprotein M (ApoM) is a newly discovered apolipoprotein with a molecular weight of 26 kDa (Xu and Dahlbäck, 1999), which is mainly expressed in liver, kidney, adipose tissue, and cerebrovascular endothelial cells of human beings (Xu and Dahlbäck, 1999; Kober et al., 2017; Sramkova et al., 2019). To date, the studies of ApoM mainly focused on lipid metabolism (Wolfrum et al., 2005), cardiovascular disease (Versmissen et al., 2016), and diabetes (Kurano et al., 2020). Of note, a genome-wide association study (GWAS) found that the *APOM* gene could drive the association of lipid metabolism pathway with AD risk (Kunkle et al., 2019; Chernick et al., 2020). Besides, the CSF level of ApoM in the patients with AD was significantly lower than that in the cognitively normal (CN) controls (Khoonsari et al., 2016). But, the relationship between blood ApoM and AD has not been revealed so far.

In addition, there are often obviously individual differences in the absolute values of certain blood-based biomarkers in clinical practice, which limits their interpretation and applications. Thus, standardization or normalization by calculating with other related clinical indicators, such as the international normalized ratio (INR) generated from the prothrombin time (PT), may lead to better stability and comparability (Deitcher, 2002). Actually, the combination of biomarkers has also been a common strategy in AD research, such as Aβ42/Aβ40 ratio, p-tau/total-tau ratio, and total-tau/α-synuclein ratio (Shim et al., 2020), etc. Considering that ApoM is closely related to lipid metabolism and based on our previous research experience (Yang et al., 2021), the ratios of blood ApoM level to that of different blood lipids including total cholesterol (TC), triglyceride (TG), high-density lipoprotein cholesterol (HDL-C), and low-density lipoprotein cholesterol (LDL-C), may be the more optimized indicators than ApoM itself (Yang et al., 2021).

Therefore, here, we reported a multicenter, cross-sectional pilot clinical study, in which AD group and CN control group were recruited. The demographic data, clinical characteristics, and some of the common indicators in clinical laboratories were collected. Then, we detected and compared the plasma ApoM levels of both groups. Further, we investigated the correlation between plasma ApoM with the presence, main clinical characteristics, and the representative biomarkers of AD, respectively. Finally, we explored the diagnostic power of plasma ApoM for AD. We expected that this study could provide new evidence for evaluating plasma ApoM as a new candidate biomarker of AD.

## Materials and Methods

### Study Design and Participant Selection

Both groups were recruited from the Department of Neurology and the Department of Gerontology of Sichuan Provincial People’s Hospital, Western Theater Command General Hospital, and the Eighth People’s Hospital of Chengdu from March to September, 2021. The diagnosis of AD was in accordance with the criteria for “Probable AD” of the National Institute of Neurological and Speech Disorders and Stroke/Alzheimer’s Disease and Related Disorders Association criteria (NINCDS-ADRDA) (McKhann et al., 1984). These procedures were administered by two trained interviewers who were experienced neurologists. The participants were excluded if they had (Zhuang et al., 2018; Zhang et al., 2021) (1) a family history of dementia; (2) severe cardiac, pulmonary, hepatic, renal diseases, or any kinds of tumor; (3) enduring mental illness (e.g., schizophrenia); and (4) other kinds of cognitive deficits, such as vascular dementia, dementia with Lewy bodies, frontotemporal dementia, dementia of Parkinson’s disease, and normal pressure hydrocephalus. We first screened the cognitive state of participants using the China version of the Mini–Mental State Examination (MMSE) and Montreal Cognitive Assessment (MoCA). The cutoff values after correcting the years of education were used in the MMSE (≤24 for 6 + years of education, ≤20 for 6 years of education, and ≤17 for 0 year of education) and MoCA (<26 for 12 + years of education and <25 for 12 years of education) (Hu et al., 2020). ADL scale was used to evaluate the daily living ability of all the participants. The full score was 56 points, and the higher the score was, the more severe the impairment of daily living ability was. The vascular factors were assessed by the Hachinski Ischemic Score (HIS). Participants with abnormal cognitive function assessment were further assessed with the Clinical Dementia Rating (CDR) in which patients with AD had a CDR score ≥0.5 (Morris, 1993).

Baseline data on demographic characteristics (age, sex, and years of education), body mass index (BMI), lifestyle risk factors (cigarette smoking and alcohol consumption), and medical history (hypertension and diabetes mellitus) were collected within 24 h of hospital admission.

### Biochemical and Molecular Investigations

Following our previously published protocols (Yang et al., 2021), fasting blood samples were collected between 06:00 and 07:00 a.m. within 24 h of admission to avoid variations related to possible circadian rhythm effects, and aliquots were made for routine blood tests [i.e., hemoglobin A1C (HbA1C), white blood cell (WBC) count, C-reactive protein (CRP), TC, TG, HDL-C, LDL-C], and *APOE* genotype [determined using a Sanger sequencing assay for single-nucleotide polymorphism (SNP) rs7412 and rs429358 (Belloy et al., 2019)] in the Department of Clinical Laboratory.

The plasma was separated and frozen at –80°C until ApoM, p-tau217, and NfL measurement. In accordance with our previous published studies (Sun et al., 2020), the plasma p-tau217 and NfL were quantified using enzyme-linked immunosorbent assay (ELISA) kits (Enzyme Biosystems). The specimen, standard, and HRP-labeled detection antibodies corresponding to two specific proteins were sequentially added to microwells precoated with human p-tau217 and NfL capture antibody, respectively. The microwells were incubated and washed thoroughly. The substrate TMB was used to develop color. TMB was first converted to a blue color under the catalysis of peroxidase and converted to the final yellow color by the action of an acid. The color depth was positively correlated with p-tau217 and NfL in the sample. Besides, we employed a commercial ELISA kit (SEC299Hu, Cloud-Clone Corp., Houston, TX, United States) to measure the plasma ApoM concentrations in accordance with the previous studies (Zhang et al., 2017). Samples and standards were measured in duplicate, and the means of the duplicates were used for statistical analysis.

### Statistical Analyses

All statistical analyses were performed using IBM SPSS Statistics version 25 (IBM, Armonk, NY, United States) and GraphPad Prism 6 (GraphPad Software, La Jolla, CA, United States). For continuous variables, data were expressed as mean ± standard deviation (SD) or median and interquartile range (IQR), which depended on the data distribution, and the Mann–Whitney *U* test or the *t*-test were used to test the differences between the two groups. Categorical variables were expressed as proportions, and a chi-square test was applied in the comparison between groups. The influence of plasma ApoM and ApoM-derived indicators on the presence of AD was determined by multivariate logistic regression analysis, which allowed adjustment for the confounding factors. Clinical variables with *p* < 0.05 on univariate analysis and the general AD risk factors (age, sex, and *APOE*4 carriage status) were incorporated into multivariate logistic regression analysis models. The results were expressed as odds ratio (OR) with corresponding 95% confidence interval (CI). Correlations between plasma ApoM and its derived indicators with clinical characteristics and the representative blood-based biomarkers of AD were analyzed using Spearman’s rank correlation or Pearson’s correlation. The clinical value of adding ApoM and ApoM-derived indicators to the general risk factors for identifying or predicting the presence of AD was calculated with receiver operating characteristic (ROC) curves. Using the DeLong’s test, we compared the area under the curve (AUC) between ApoM with ApoM-derived indicators and between the basic model (sex, age, *APOE*4, and BMI) with basic model combined with ApoM and its derived indicators, respectively. All tests were two-sided, and *p* < 0.05 was considered statistically significant. Goodness-of-fit of logistic regression models was assessed using the Hosmer–Lemeshow test. None of the above models displayed a Hosmer–Lemeshow chi-squared value yielding a *p* < 0.05, and therefore, none were rejected.

## Results

### Comparison of Baseline Data Between Two Groups

Eventually, a total of 140 participants were recruited including CN group (*n* = 73) and AD group (*n* = 67), and the detailed enrollment process for participants is shown in Supplementary Figure 1. The demographic characteristics, BMI, lifestyle risk factors, medical history, cognitive function, ADL, and blood tests of all participants are shown in Table 1. No differences in age, sex, years of education, cigarette smoking, alcohol consumption, hypertension, diabetes mellitus, WBC count, HbA1c, and CRP between two groups were found (each *p* > 0.05) (Table 1). Compared with CN group, the percentage of *APOE*4-positive was significantly higher in AD group (12.3 vs. 38.3%, *p* < 0.0001), whereas BMI (23.86 ± 2.99 vs. 20.72 ± 2.19, *p* < 0.0001), TC (4.20 ± 0.86 vs. 3.76 ± 0.72, *p* = 0.001), TG (1.68 ± 0.54 vs. 1.24 ± 0.41, *p* < 0.0001), and HDL-C (1.33 ± 0.29 vs. 1.18 ± 0.24, *p* = 0.001) were markedly lower, respectively.

**TABLE 1:** Demographics, clinical characteristics, and blood tests of all participants.

Parameters	CN group (*n* = 73)	AD group (*n* = 67)	*t/x^2^/Z*	*p*-value
Age (years), mean (SD)	76.96 (4.56)	78.70 (5.90)	−1.963	0.052
Sex, female, No. (%)	39 (53.4)	39 (58.2)	0.324	0.569
Years of education (years), median (IQR)	9 (9–12)	9 (6–12)	−1.598	0.110
*APOE* ε4-positive, No. (%)	9 (12.3)	26 (38.8)	13.062	<0.0001
BMI, mean (SD), kg/m^2^	23.86 (2.99)	20.72 (2.19)	7.041	<0.0001
Cigarette smoking, No. (%)	22 (30.1)	18 (26.9)	0.183	0.669
Alcohol consumption, No. (%)	21 (28.8)	15 (22.4)	0.744	0.388
Diabetes, No. (%)	28 (38.4)	21 (31.3)	0.755	0.385
Hypertension, No. (%)	51 (69.9)	47 (70.1)	0.001	0.971
HbA1c, median (IQR), (%)	5.90 (5.52–6.40)	5.70 (5.50–6.40)	−0.480	0.631
WBC, mean (SD, (×10^9^/L)	6.34 (1.66)	6.23 (1.70)	0.401	0.689
CRP, median (IQR), (mg/L),	1.17 (0.62–2.61)	1.43 (0.71–3.70)	−0.880	0.379
TC, mean (SD), (mmol/L)	4.20 (0.86)	3.76 (0.72)	3.294	0.001
TG, mean (SD, (mmol/L)	1.68 (0.54)	1.24 (0.41)	5.356	<0.0001
HDL-C, mean (SD), (mmol/L)	1.33 (0.29)	1.18 (0.24)	3.387	0.001
LDL-C, median (IQR), (mmol/L)	2.24 (1.70–2.96)	2.06 (1.71–2.53)	−1.827	0.068
MMSE score, median (IQR)	28 (27–29)	10 (8–14)	−10.240	<0.0001
MoCA score, median (IQR)	25 (25–26)	6 (4–8)	−10.361	<0.0001
ADL score, median (IQR)	14 (14–14)	43 (31–52)	−10.878	<0.0001
CDR global (0/0.5/1/2/3)	79/0/0/0/0	0/0/19/32/16	/	/
CDR-SB, median (IQR)	0	11.00 (8.00–14.00)	−11.017	<0.0001
p-tau217, mean (SD), (pg/mL)	174.14 (89.20)	276.31 (128.37)	−5.423	<0.0001
NfL, mean (SD), (pg/mL)	532.64 (189.28)	851.93 (344.70)	−6.710	<0.0001
ApoM, median (IQR), (μg/mL)	52.66 (41.20–61.44)	64.24 (50.72–82.85)	−4.634	<0.0001
ApoM/TC ratio, median (IQR)	12.29 (9.49–15.52)	19.17 (13.55–23.34)	−5.771	<0.0001
ApoM/TG ratio, median (IQR)	30.26 (24.62–42.24)	55.20 (41.61–77.64)	−6.622	<0.0001
ApoM/HDL-C ratio, mean (SD)	40.43 (14.46)	61.85 (24.97)	−6.139	<0.0001
ApoM/LDL-C ratio, median (IQR)	21.50 (16.52–32.19)	30.48 (24.24–42.01)	−4.319	<0.0001

*CN, cognitively normal; AD, Alzheimer’s disease; SD, standard deviation; IQR, interquartile range; APOE, apolipoprotein E; BMI, body mass index; HbA1c, hemoglobin A1c; WBC, white blood cell; CRP, C-reactive protein; TC, total cholesterol; TG, triglyceride; HDL-C, high-density lipoprotein cholesterol; LDL-C, low-density lipoprotein cholesterol; MMSE: Mini–Mental State Examination; MoCA, Montreal Cognitive Assessment; ADL, activities of daily living; CDR, Clinical Dementia Rating; CDR-SB, Clinical Dementia Rating Sum of Boxes; p-tau217, tau phosphorylated at threonine 217; NfL: neurofilament light chain; ApoM, apolipoprotein M. p < 0.05 was considered the statistical significance.*

**FIGURE 1:**
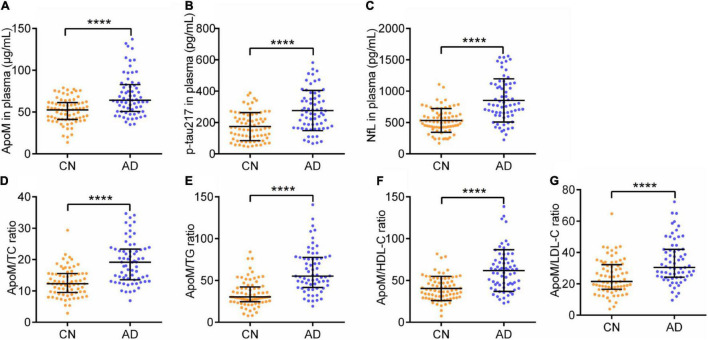
Plasma levels of ApoM, p-tau217, NfL, and ApoM-derived indicators between two groups. ApoM, apolipoprotein M; p-tau217, tau phosphorylated at threonine 217; NfL, neurofilament light chain; CN, cognitively normal; AD, Alzheimer’s disease; TC, total cholesterol; TG, triglyceride; HDL-C, high-density lipoprotein cholesterol; LDL-C, low-density lipoprotein cholesterol; SD, standard deviation; IQR, interquartile range. Median with IQR **(A,D,E,G)** and mean ± SD **(B,C,F)**. Statistical comparison was performed using Mann–Whitney *U* test **(A,D,E,G)** or Student’s *t*-test **(B,C,F)**. ****p ≤ 0.0001. *p* < 0.05 was considered the statistical significance.

As for cognitive function and ADL, AD group had significantly lower MMSE scores [28 (27–29) vs. 10 (8–14), *p* < 0.0001] and MoCA scores [25 (25–26) vs. 6 (4–8), *p* < 0.0001], and remarkably higher ADL scores [14 (14–14) vs. 43 (31–52), *p* < 0.0001] than CN group, respectively (Table 1). Regarding the representative blood biomarkers, plasma levels of p-tau 217 (174.14 ± 89.20 vs. 276.31 ± 128.37, *p* < 0.0001) and NfL (532.64 ± 189.28 vs. 851.93 ± 344.70, *p* < 0.0001) in AD group were significantly higher than those in CN group, respectively (Table 1 and Figure 1).

Alzheimer’s disease group had a significantly higher plasma ApoM level [52.66 (41.20–61.44) vs. *p* < 0.0001] than CN group (Table 1 and Figure 1). Besides, each of the ApoM-derived indicators that include the ratios of ApoM/TC [12.29 (9.49–15.52) vs. 19.17(13.55–23.34), *p* < 0.0001], ApoM/TG [30.26 (24.62–42.24) vs. 55.20 (41.61–77.64), *p* < 0.0001], ApoM/HDL-C (40.43 ± 14.46 vs. 61.85 ± 24.97, *p* < 0.0001), and ApoM/LDL-C [21.50 (16.52–32.19) vs. 30.48 (24.24–42.01), *p* < 0.0001] was significantly higher in AD group than that in CN group (Table 1 and Figure 1).

### Association Between Plasma Apolipoprotein M With the Presence of Alzheimer’s Disease

The associations of plasma ApoM level and its derived indicators with the presence of AD were investigated by multivariate logistic regression analysis. When AD risk factors (age, sex, and *APOE*4 carriage status) and BMI were adjusted, the plasma ApoM level (OR = 1.058, 95% CI: 1.027–1.090, *p* < 0.0001) and the ratios of ApoM/TC (OR = 1.239, 95% CI: 1.120–1.372, *p* < 0.0001), ApoM/TG (OR = 1.064, 95% CI: 1.035–1.095, *p* < 0.0001), ApoM/HDL-C (OR = 1.069, 95% CI: 1.037–1.102, *p* < 0.0001), and ApoM/LDL-C (OR = 1.064, 95% CI: 1.023–1.106, *p* = 0.002) were significantly associated with the presence of AD, respectively (Table 2).

**TABLE 2:** Independent factors associated with the presence of AD in binary logistic regression.

	Parameters	OR value (95% CI)	*p*-value
	*APOE* ε4-positive	4.271(1.462–12.478)	0.008
Model 1	BMI	0.584(0.463–0.737)	<0.0001
	ApoM	1.058(1.027–1.090)	<0.0001
	*APOE* ε4-positive	4.676(1.548–14.125)	0.006
Model 2	BMI	0.604(0.481–0.758)	<0.0001
	ApoM/TC ratio	1.239(1.120–1.372)	<0.0001
	*APOE* ε4-positive	3.605(1.203–10.803)	0.022
Model 3	BMI	0.626(0.499–0.787)	<0.0001
	ApoM/TG ratio	1.064(1.035–1.095)	<0.0001
	*APOE* ε4-positive	4.519(1.515–13.486)	0.007
Model 4	BMI	0.588(0.466–0.741)	<0.0001
	ApoM/HDL-C ratio	1.069(1.037–1.102)	<0.0001
	*APOE* ε4-positive	3.965(1.393–11.284)	0.010
Model 5	BMI	0.603(0.487–0.747)	<0.0001
	ApoM/LDL-C ratio	1.064(1.023–1.106)	0.002

*Model 1: age, sex, APOE ε4-positive, BMI, and ApoM.*

*Model 2: age, sex, APOE ε4-positive, BMI, and ApoM/TC ratio.*

*Model 3: age, sex, APOE ε4-positive, BMI, and ApoM/TG ratio.*

*Model 4: age, sex, APOE ε4-positive, BMI, and ApoM/HDL-C ratio.*

*Model 5: age, sex, APOE ε4-positive, BMI, and ApoM/LDL-C ratio.*

*OR, odds ratio; CI, confidence interval; APOE, apolipoprotein E; BMI, body mass index; ApoM, apolipoprotein M; TC, total cholesterol; TG, triglyceride; HDL-C, high-density lipoprotein cholesterol; LDL-C, low-density lipoprotein cholesterol. p < 0.05 was considered the statistical significance.*

### Correlations Between Plasma Apolipoprotein M With Clinical Characteristics of Alzheimer’s Disease

**FIGURE 2:**
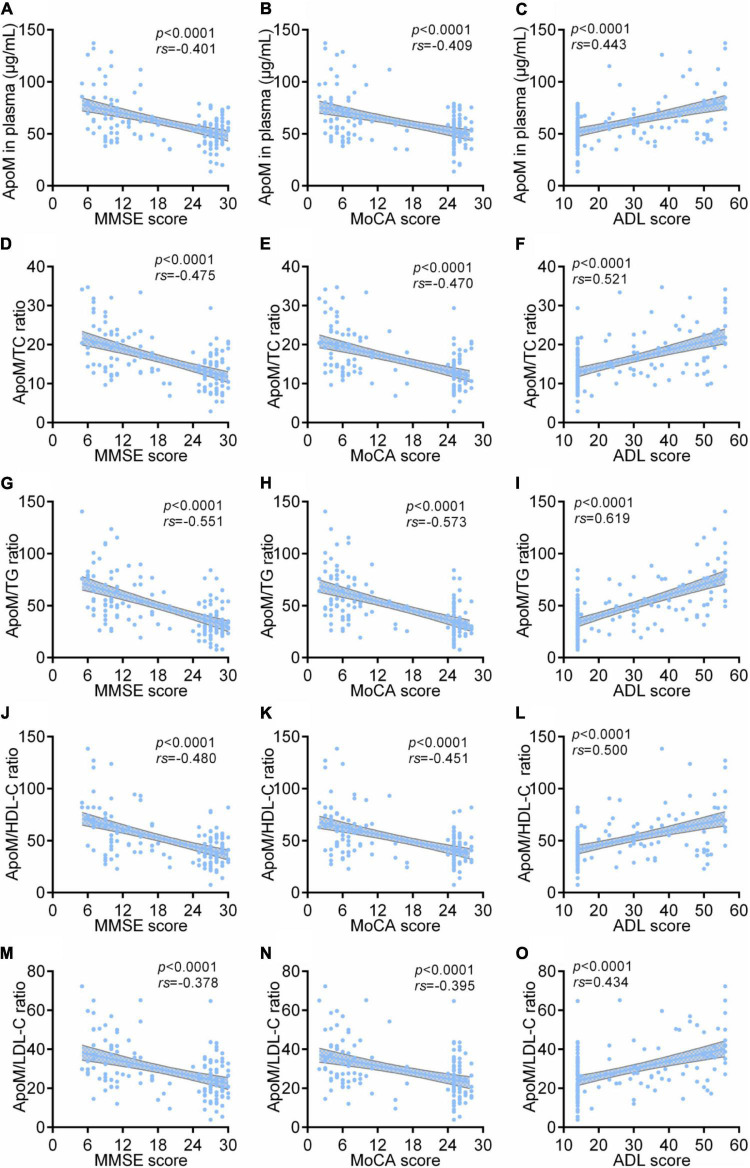
Correlations between plasma ApoM and its derived indicators with clinical characteristics of AD among all participants. The correlations between plasma ApoM with MMSE score **(A)**, MoCA score **(B)** and ADL score **(C)**. The correlations between ApoM/TC ratio with MMSE score **(D)**, MoCA score **(E)** and ADL score **(F)**. The correlations between ApoM/TG ratio with MMSE score **(G)**, MoCA score **(H)** and ADL score **(I)**. The correlations between ApoM/HDL-C ratio with MMSE score **(J)**, MoCA score **(K)** and ADL score **(L)**. The correlations between ApoM/LDL-C ratio with MMSE score **(M)**, MoCA score **(N)** and ADL score **(O)**. ApoM, apolipoprotein M; AD, Alzheimer’s disease; MMSE, Mini–Mental State Examination; MoCA, Montreal Cognitive Assessment; ADL, activities of daily living; TC, total cholesterol; TG, triglyceride; HDL-C, high-density lipoprotein cholesterol; LDL-C, low-density lipoprotein cholesterol. Correlation analyses were performed using Spearman’s rank correlation. *P* < 0.05 was considered the statistical significance.

Among all participants, ApoM and four derived indicators were significantly negatively correlated with MMSE scores and MoCA scores and significantly positively correlated with ADL scores, respectively (each *p* < 0.0001). Thereinto, ApoM/TG ratio had the highest absolute value of correlation coefficient in each analysis (*rs* = –0.551 with MMSE scores, *rs* = –0.573 with MoCA scores, *rs* = 0.619 with ADL scores) (Figure 2).

**FIGURE 3:**
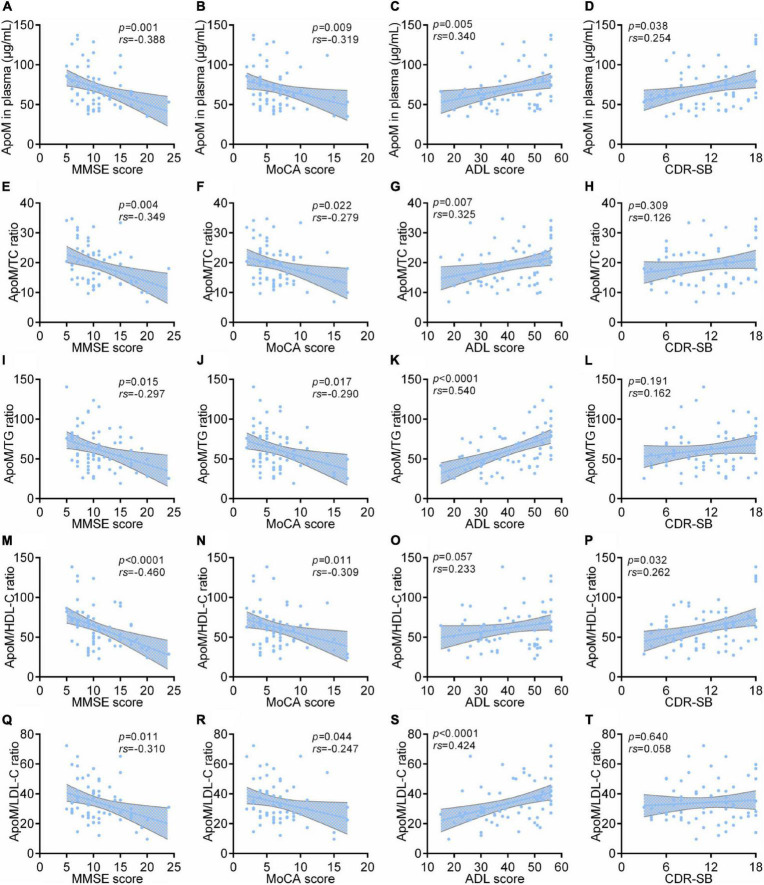
Correlations between plasma ApoM and its derived indicators with clinical characteristics of AD in AD group. The correlations between plasma ApoM with MMSE score **(A)**, MoCA score **(B)**, ADL score **(C)** and CDR-SB **(D)**. The correlations between ApoM/TC ratio with MMSE score **(E)**, MoCA score **(F)**, ADL score **(G)** and CDR-SB **(H)**. The correlations between ApoM/TG ratio with MMSE score **(I)**, MoCA score **(J)**, ADL score **(K)** and CDR-SB **(L)**. The correlations between ApoM/HDL-C ratio with MMSE score **(M)**, MoCA score **(N)**, ADL score **(O)** and CDR-SB **(P)**. The correlations between ApoM/LDL-C ratio with MMSE score **(Q)**, MoCA score **(R)**, ADL score **(S)** and CDR-SB **(T)**. ApoM, apolipoprotein M; AD, Alzheimer’s disease; MMSE, Mini–Mental State Examination; MoCA, Montreal Cognitive Assessment; ADL, activities of daily living; CDR-SB, Clinical Dementia Rating Sum of Boxes; TC, total cholesterol; TG, triglyceride; HDL-C, high-density lipoprotein cholesterol; LDL-C, low-density lipoprotein cholesterol. Correlation analyses were performed using Spearman’s rank correlation. *p* < 0.05 was considered the statistical significance.

In AD group, ApoM and all the derived indicators were significantly negatively correlated with MMSE scores and MoCA scores, respectively (each *p* < 0.05), but each absolute value of correlation coefficient was low (<0.5). Except for ApoM/HDL-C ratio, ApoM and the other indicators were significantly positively correlated with ADL scores (each *p* < 0.01), in which ApoM/TG ratio had the highest correlation coefficient (*rs* = 0.540). Furthermore, we found that plasma ApoM and ApoM/HDL-C ratio were significantly positively correlated with the CDR sum of boxes (CDR-SB) total scores (Figure 3).

We performed the subgroup analyses of all participants according to their *APOE* 4 carriage status and sex, respectively. Among *APOE* 4-negative participants (*n* = 105), ApoM and all the derived indicators were significantly negatively correlated with MMSE scores (each *p* < 0.01) and MoCA scores (each *p* < 0.0001), and markedly positively correlated with ADL scores (each *p* < 0.0001), respectively (Supplementary Figure 2). ApoM/TG ratio performed best in the analysis with MMSE scores (*rs* = –0.540) and with MoCA scores (*rs* = –0.569). Both ApoM/TG ratio (*rs* = 0.585) and ApoM/TC ratio (*rs* = 0.507) performed better in the analysis with ADL scores (Supplementary Figure 2). Moreover, the trend of each correlation analysis results in *APOE* 4-positive participants (*n* = 35) was consistent with that of *APOE* 4-negative of statistical significance (each *p* < 0.05) (Supplementary Figure 2). In detail, the ratios of ApoM/TC (*rs* = –0.553), ApoM/HDL-C (*rs* = –0.534), and ApoM/LDL-C (*rs* = –0.529) performed better in the analysis with MMSE scores, the ApoM/TC ratio got the best correlation coefficient in the analysis with MoCA scores (*rs* = –0.522), and the ratios of ApoM/TC (*rs* = 0.537), ApoM/TG (*rs* = 0.572), and ApoM/LDL-C (*rs* = 0.578) had better correlations with ADL scores (Supplementary Figure 2). In addition, we conducted subgroup analyses by sex. Interestingly, the correlation between ApoM and four derived indicators with clinical characteristics of AD still remained (Supplementary Figure 3).

### Correlations Between Plasma Apolipoprotein M With Representative Blood Biomarkers of Alzheimer’s Disease

**FIGURE 4:**
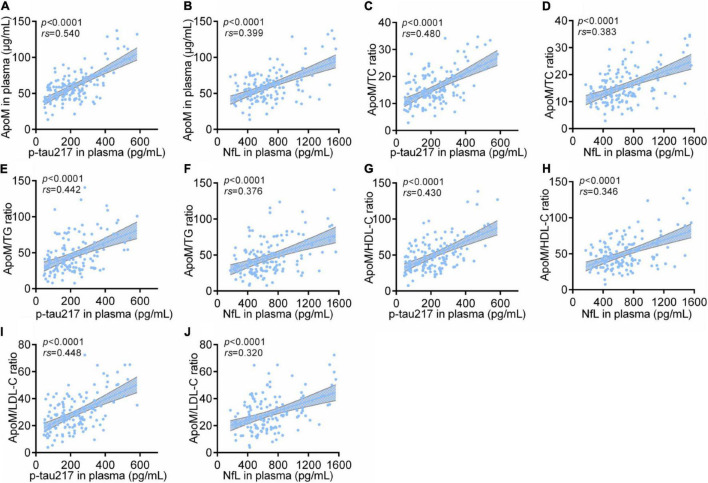
Correlations between plasma ApoM and its derived indicators with representative blood biomarkers of AD among all participants. The correlations between plasma ApoM with plasma p-tau217 **(A)** and NfL **(B)**. The correlations between ApoM/TC ratio with plasma p-tau217 **(C)** and NfL **(D)**. The correlations between ApoM/TG ratio with plasma p-tau217 **(E)** and NfL **(F)**. The correlations between ApoM/HDL-C ratio with plasma p-tau217 **(G)** and NfL **(H)**. The correlations between ApoM/LDL-C ratio with plasma p-tau217 **(I)** and NfL **(J)**. ApoM, apolipoprotein M; p-tau217, tau phosphorylated at threonine 217; NfL, neurofilament light chain; CN, cognitively normal; AD, Alzheimer’s disease; TC, total cholesterol; TG, triglyceride; HDL-C, high-density lipoprotein cholesterol; LDL-C, low-density lipoprotein cholesterol. Correlation analysis was performed with Spearman’s rank correlation. *P* < 0.05 was considered the statistical significance.

**FIGURE 5:**
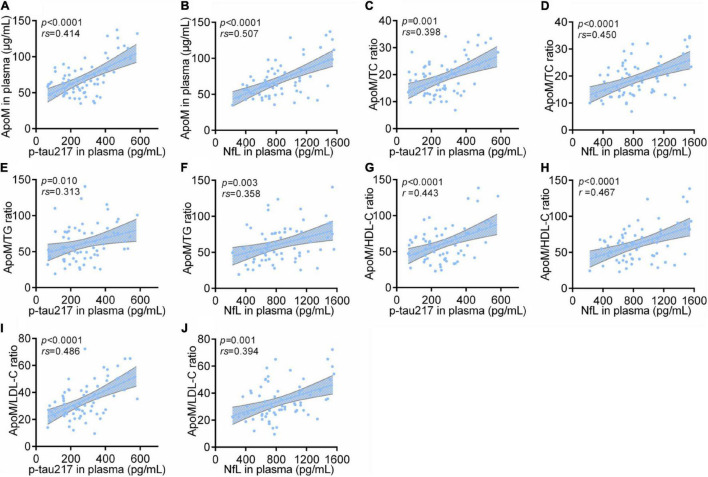
Correlations between plasma ApoM and its derived indicators with representative blood biomarkers of AD in AD group. ApoM, apolipoprotein M; p-tau217, tau phosphorylated at threonine 217; NfL, neurofilament light chain; CN, cognitively normal; AD, Alzheimer’s disease; TC, total cholesterol; TG, triglyceride; HDL-C, high-density lipoprotein cholesterol; LDL-C, low-density lipoprotein cholesterol. Correlation analyses were performed using Spearman’s rank correlation **(A–F,I,J)** or Pearson’s correlation **(G,H)** as appropriate. *P* < 0.05 was considered the statistical significance.

Among all participants, ApoM and four derived indicators were significantly positively correlated with plasma levels of p-tau217 and NfL, respectively (each *p* < 0.0001), in which ApoM had the highest correlation coefficient in the analysis with p-tau217 (*rs* = 0.540), but each of the other correlation coefficient was low (<0.5) (Figure 4). In AD group, ApoM and all the derived indicators were significantly positively correlated with plasma p-tau217 (each *p* ≤ 0.01) and NfL (each *p* ≤ 0.001), but all the correlation coefficients were low (<0.5) except that of ApoM with NfL (*rs* = 0.507) (Figure 5).

The trend of each correlation analysis results among *APOE* 4-negative participants (*n* = 105) was consistent with that in AD group of statistical significance (each *p* < 0.0001 in analysis with p-tau217 and each *p* < 0.05 in analysis with NfL) (Supplementary Figure 4). In detail, all the correlation coefficients were low (<0.5) except that of ApoM with p-tau217 (*rs* = 0.535) (Supplementary Figure 4). Except for ApoM/HDL-C ratio, the other indicators had the same statistical trend among *APOE* 4-positive participants (*n* = 35) with p-tau217 (each *p* ≤ 0.01) and NfL (each *p* ≤ 0.05), respectively (Supplementary Figure 4). Specifically, the ratios of ApoM/TG (*rs* = 0.500) and ApoM/LDL-C (*rs* = 0.525) performed better in the analysis with p-tau217, and ApoM/LDL-C ratio got the highest correlation coefficient in the analysis with NfL (*rs* = 0.525) (Supplementary Figure 4). Moreover, we conducted subgroup analyses by sex. The correlations between ApoM and four derived indicators representative blood biomarkers of AD still remained (Supplementary Figure 5).

### The Diagnostic Power of Plasma Apolipoprotein M for Alzheimer’s Disease

**FIGURE 6:**
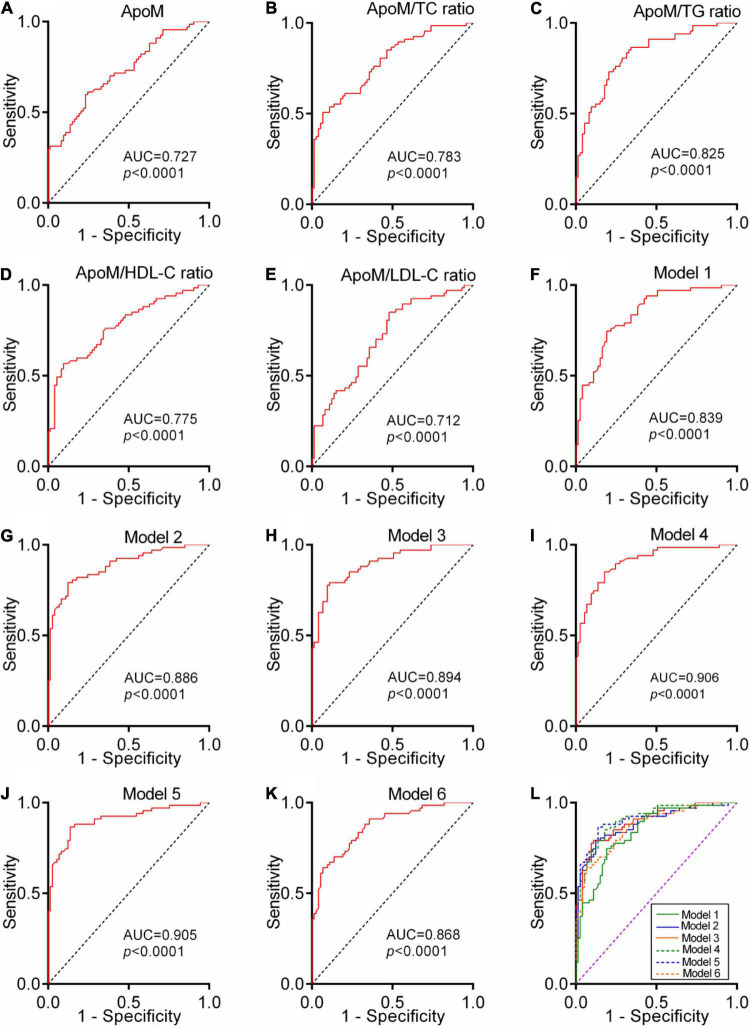
Diagnostic power of plasma ApoM and its derived indicators for AD. ROC analyses of ApoM **(A)**, ApoM/TC ratio **(B)**, ApoM/TG ratio **(C)**, ApoM/HDL-C ratio **(D)** and ApoM/LDL-C ratio **(E)**. Model 1: age, sex, *APOE* ε4, and BMI **(F)**. Model 2: age, sex, *APOE* ε4, BMI, and ApoM **(G)**. Model 3: age, sex, *APOE* ε4, BMI, and ApoM/TC ratio **(H)**. Model 4: age, sex, *APOE* ε4, BMI, and ApoM/TG ratio **(I)**. Model 5: age, sex, *APOE* ε4, BMI, and ApoM/HDL-C ratio **(J)**. Model 6: age, sex, *APOE* ε4, BMI, and ApoM/LDL-C ratio **(K)**. Comparison of ROC-AUCs of all the models using DeLong’s test **(L)**. ApoM, apolipoprotein M; AD, Alzheimer’s disease; TC, total cholesterol; TG, triglyceride; HDL-C, high-density lipoprotein cholesterol; LDL-C, low-density lipoprotein cholesterol, BMI, body mass index; APOE, apolipoprotein E; ROC, receiver operating characteristic; AUC, area under the curve. *P* < 0.05 was considered the statistical significance.

To analyze the performance of plasma ApoM and its derived indicators to distinguish patients with AD from CN controls, we performed a ROC analysis. Each indicator featured significantly high AUC, which far exceeded the random chance (AUC of 50%) (each *p* < 0.0001, AUCs ranged from 0.712 to 0.825) (Figure 6). DeLong’s test was used to compare the ROC curve of these five indicators mutually. The ApoM/TC ratio (ΔAUC = 0.056, *p* = 0.044) and ApoM/TG ratio (ΔAUC = 0.097, *p* = 0.011) had a statistically remarkably larger AUC than ApoM, respectively (Table 3).

**TABLE 3:** Pairwise comparison of the area under the receiver operator characteristic curves by the DeLong’s test.

	Difference between areas	*Z* statistic	*p*-value
ApoM vs. ApoM/TC ratio	0.056	2.010	0.044
ApoM vs. ApoM/TG ratio	0.097	2.533	0.011
ApoM vs. ApoM/HDL-C ratio	0.048	1.814	0.070
ApoM vs. ApoM/LDL-C ratio	0.015	0.382	0.702
ApoM/TC ratio vs. ApoM/TG ratio	0.042	1.079	0.2808
ApoM/TC ratio vs. ApoM/HDL-C ratio	0.008	0.254	0.799
ApoM/TC ratio vs. ApoM/LDL-C ratio	0.071	2.564	0.010
ApoM/TG ratio vs. ApoM/HDL-C ratio	0.049	1.159	0.246
ApoM/TG ratio vs. ApoM/LDL-C ratio	0.113	2.439	0.015
ApoM/HDL-C ratio vs. ApoM/LDL-C ratio	0.063	1.560	0.119

*ApoM, apolipoprotein M; TC, total cholesterol; TG, triglyceride; HDL-C, high-density lipoprotein cholesterol; LDL-C, low-density lipoprotein cholesterol. P < 0.05 was considered the statistical significance.*

A ROC curve constructed by combining age, sex, *APOE* 4, and BMI got a significantly larger AUC than the random chance (Model 1, *p* < 0.0001, AUC = 0.839). The independent addition of ApoM and its derived indicators to Model 1 established Model 2–6 with a significantly larger AUC than the random chance, respectively (each *p* < 0.0001, AUCs ranged from 0.868 to 0.906) (Figure 6). DeLong’s test showed that Model 2–5 had statistically higher diagnostic power than Model 1, respectively (each *p* < 0.05) (Supplementary Table 1).

## Discussion

In this multicenter, cross-sectional study, we found for the first time that plasma level of ApoM and its derived indicators (i.e., ratios of ApoM/TC, ApoM/TG, ApoM/HDL-C, and ApoM/LDL-C) were significantly higher in AD group than those in CN group, respectively. Then, we confirmed that plasma ApoM and its derived indicators were remarkably associated with the presence of AD, respectively. Subsequently, the correlations between plasma ApoM and its derived indicators with clinical characteristics (i.e., cognitive impairment and ADL) and representative blood biomarkers (i.e., p-tau217 and NfL) of AD were analyzed, respectively. Finally, we assessed the diagnostic power of plasma ApoM for AD and revealed that the majority of the four derived indicators performed individually better than ApoM. Meanwhile, the plasma ApoM and all the derived indicators could significantly enhance the diagnostic efficiency of the basic model of combining age, sex, *APOE*4 carriage status, and BMI, respectively. To our knowledge, this is the first time to reveal the association between plasma ApoM with AD so far.

In the past decades, the early diagnosis and the exploration of biomarkers have been the frontiers of AD research. The research framework proposed by NIA-AA in 2018 categorized the biomarkers of AD as A/T(N), where A represents Aβ deposition, T represents tau hyperphosphorylation and neurofibrillary tangle (NFT) formation, and N represents neuronal loss and neurodegeneration (Jack et al., 2018). This framework requires that the establishment of AD definitive diagnosis must be based on the evidence of brain Aβ aggregation (cerebrospinal fluid or PET test) (Jack et al., 2018). However, the high cost of invasive lumbar puncture and molecular imaging greatly limit their applications. Thus, blood-derived biomarkers are attracting increasing attention from clinicians and researchers (Thijssen and Rabinovici, 2021).

At present, both plasma p-tau217 and NfL are representative among all blood-based biomarkers of AD (Janelidze et al., 2021b). Additionally, a series of novel blood biomarkers and combinations of different biomarkers were also being developed and validated taking plasma p-tau217 and NfL as reference (Cullen et al., 2021; Palmqvist et al., 2021a). Thus, in this study, we measured the plasma levels of p-tau217 and NfL and further analyzed their correlations with ApoM and its derived indicators, respectively. All the results from total participants and subgroup analyses (sex and *APOE*4 carriage status) indicated that plasma ApoM had the potential to evaluate the severity of neurodegeneration of AD. The extensive studies on plasma p-tau217 have shown an excellent accuracy of p-tau217 in differentiating patients with AD and non-AD (Palmqvist et al., 2020; Palmqvist et al., 2021b; Thijssen et al., 2021). However, in this study, we found an overlap of plasma p-tau217 between the AD and CN groups. The previous studies have shown that biomarkers may vary with the stage and severity of AD, and the CSF p-tau as an early biomarker of downstream pathological changes was triggered by Aβ deposition (Sato et al., 2018). Research has shown different patterns of changes in CSF p-tau and tau-PET measures across the AD continuum. CSF levels of p-tau increased in the earliest disease stages (i.e., in asymptomatic individuals with CSF or PET evidence of abnormal Aβ accumulation) and appeared to reach a plateau or even decrease in later symptomatic stages of AD (Fagan et al., 2014; Mattsson-Carlgren et al., 2020a; Janelidze et al., 2021a). Considering the consistency of CSF and plasma p-tau (Barthélemy et al., 2020; Palmqvist et al., 2020; Janelidze et al., 2021a), it is speculated that the plasma p-tau217 may have a similar trajectory to CSF p-tau217. Most of the patients with AD in previous clinical studies involving plasma p-tau217 were at the stage of mild or moderate AD (Mattsson-Carlgren et al., 2020b,^2021^). However, many patients with AD in this study were moderate to severe AD (CDR scored 2–3), which indicates that the plasma p-tau217 might have entered a plateau or even declined, which could result in an overlap with CN group.

However, the role and related mechanism of ApoM in the pathogenesis of AD is uncovered. Reviewing the previously published literature, we proposed that ApoM might influence on Aβ metabolism and AD pathogenesis through the pathways as followings. First, sphingosine 1-phosphate (S1P), a metabolite of membrane sphingosine lipid, binds to the extracellular partners, is enriched in circulating fluid, and binds to G protein-coupled S1P receptors (S1PRs), which regulates the embryonic development and organ function (Cartier and Hla, 2019). It showed that immunoregulatory S1P had the potential as a biomarker for plasma diagnosis and differential diagnosis of AD (Chua et al., 2020). Postmortem study found that the level of brain S1P was reduced in patients with AD and significantly negatively correlated with the level of brain Aβ and hyperphosphorylated tau protein (He et al., 2010). Cell experiments showed that S1P was specifically bound to full-length BACE1 and increased its proteolytic activity. Reducing S1P generation in mouse neurons could reduce BACE1 activity level and Aβ production (Takasugi et al., 2011). It was also found that the accumulation of intracellular S1P disrupted the amyloid precursor protein (APP) metabolism. Inhibition of S1P lyase led to the accumulation of S1P, which impeded lysosomal degradation of APP, that leads to the accumulation of APP in cells and increase of APP amyloid fragment, and also reduced γ-secretase activity (Karaca et al., 2014). Nevertheless, in some neurodegenerative models, S1P has been shown to counteract the pro-apoptotic effect of neuramide by reducing oxidative stress and regulating the expression of pro-apoptotic and anti-apoptotic proteins in the Bcl-2 family (Czubowicz and Strosznajder, 2014). It was found that ApoM-bound S1P maintained the low level of permeability of paracellular blood–brain barrier (BBB) in all cerebral microvessels and low level of vesicle-mediated transport in the penetrating arterioles (Mathiesen Janiurek et al., 2019). In addition, S1P receptor 1 of cerebral endothelial cells maintained the BBB permeability in a size-selective manner by regulating the correct localization of tight junction protein (Yanagida et al., 2017). In addition, accumulation of S1P in neurons activated the microglia through S1P receptor 2, which leads to impaired autophagy and neuroinflammation (Karunakaran et al., 2019). The S1P axis in the nucleus might promote the transformation of microglia from M1 to M2 phenotype, thus regulating the neuroinflammation (Ji et al., 2019). Therefore, considering that ApoM is an important molecular chaperone of S1P, it stably binds and transports S1P in circulation and interstitial fluid, promoting the activation of receptor S1PRs and forming an important signal axis (Christoffersen et al., 2011), and ApoM is also involved in regulating the metabolism and transport of S1P (Christensen et al., 2017). These studies suggested that ApoM could regulate Aβ metabolism and AD pathogenesis through S1P and its receptors.

Second, the scavenger receptor class B type I (SR-BI) has been found in astrocytes and vascular smooth muscle cells and has been shown to mediate the adhesion of microglia to fibrillary Aβ. Microglia can directly uptake the aggregates of Aβ *via* scavenger receptors (Paresce et al., 1996). It showed that the reduction of SR-BI enhanced the deposition of fibrillary Aβ and cerebral amyloid angiopathy, impaired the response of perivascular macrophages to Aβ, and exacerbated the deficits of learning and memory (Thanopoulou et al., 2010). SR-BI keeps Aβ in a soluble state by blocking the entry of Aβ into endothelial cells, thus mediating the inhibition of Aβ-mediated monocyte adhesion to cerebrovascular endothelial cells by HDL (Robert et al., 2017). In addition, synaptic plasticity (long-term potentiation) in the CA1 region of hippocampus was found to be defective in very old SR-BI knockout mice, which also showed selective impairment in recognition and spatial memory (Chang et al., 2009). Thus, considering that ApoM is highly bound to SR-BI (Yao et al., 2020), it suggested that ApoM might regulate Aβ metabolism and AD pathogenesis through SR-BI.

Third, ApoM is widely distributed in lipoprotein in plasma, and most of it (about 95%) mainly binds to HDL, which plays a crucial role in the formation of pre-β-HDL and cholesterol efflux (Wolfrum et al., 2005). Although it is currently considered that low-level dysfunction of HDL is both risk factors for AD, the relationship and mechanism between HDL with Aβ metabolism and AD pathogenesis are still not elucidated (Stukas et al., 2014). A recent GWAS study preliminarily found that *APOM* gene was the main regulation gene of intracellular cholesterol that drove lipid metabolism pathway and thus increased the risk of AD, which suggests that ApoM may play an important role in AD (Kunkle et al., 2019). These findings suggested that ApoM could be a key in unlocking the link between HDL and AD.

Currently, ApoM is not a clinical blood lipid indicator. To improve the application value of ApoM in the clinical practice, we adopted the ratio of ApoM to clinically common blood lipid indicators based on our previous experience (Yang et al., 2021) and sought the more optimized blood biomarkers related to ApoM. The results showed that some of the four ApoM-derived indicators could perform better in their correlation analysis with cognitive function, abilities of daily living, and the representative blood biomarkers and also performed better in the diagnosis of AD compared with ApoM itself.

This study still has the following limitations: (1) As a cross-sectional clinical study, it only revealed the association between plasma ApoM and AD, but could not determine the causal relationship between them. (2) Due to the fact that most patients with AD were hospitalized for a long time and their families were not willing to undergo lumbar puncture and PET examination, the inclusion criteria for AD group in this study were based on the clinical criteria of NINCDS-ADRDA rather than the most popular biomarker-based criteria of NIA-AA. (3) The Eighth People’s Hospital of Chengdu is a chronic disease hospital and municipal sanatorium for cadres. Many patients with AD are long-term hospitalized, and a majority of them had only underwent the head CT scanning. In addition, the brain MRI data often lacked the coronal images to assess hippocampal atrophy. Thus, we did not determine the association between ApoM and neuroimaging. (4) Considering the sample size of this study, we did not carry out the external validation after diagnostic power evaluation.

Taken together, this study preliminarily uncovered that the plasma ApoM is correlated with the presence, clinical characteristics, and representative blood biomarkers of AD and has certain diagnostic power of AD. The role and related mechanism of ApoM on the pathogenesis of AD is worthy of further study in the future.

## Data Availability Statement

The raw data supporting the conclusions of this article will be made available by the authors, without undue reservation.

## Ethics Statement

The studies involving human participants were reviewed and approved by the Ethics Review Committee of Sichuan Provincial People’s Hospital. The patients/participants provided their written informed consent to participate in this study.

## Author Contributions

W-dL, S-JY, and YX carried out the study conception and design. J-YX, XH, YS, H-SJ, JF, N-wY, F-QG, FY, and JX performed the material preparation, data collection, and analysis. J-YX and YX wrote the first draft of the manuscript. All authors commented on the previous versions of the manuscript and read and approved the final manuscript.

## Conflict of Interest

The authors declare that the research was conducted in the absence of any commercial or financial relationships that could be construed as a potential conflict of interest.

## Publisher’s Note

All claims expressed in this article are solely those of the authors and do not necessarily represent those of their affiliated organizations, or those of the publisher, the editors and the reviewers. Any product that may be evaluated in this article, or claim that may be made by its manufacturer, is not guaranteed or endorsed by the publisher.

## Supplementary Material

Supplementary Figure 1 The flow diagram for the enrollment of the two groups. MMSE, mini-mental state examination; MoCA, Montreal cognitive assessment; ADL, activities of daily living; CDR-SB, Clinical Dementia Rating; CT, Computed tomography; MRI, Magnetic resonance imaging; AD, Alzheimer’s disease; CN, cognitively normal.

Supplementary Figure 2 Correlations between plasma ApoM and its-derived indicators with clinical characteristics of AD in subgroup analysis based on APOEε4 status. ApoM, apolipoprotein M; AD, Alzheimer’s disease; APOE, apolipoprotein E; MMSE, mini-mental state examination; MoCA, Montreal cognitive assessment; ADL, activities of daily living; TC, total cholesterol; TG, triglyceride; HDL-C, high-density lipoprotein cholesterol; LDL-C, low-density lipoprotein cholesterol. Correlation analyses were performed using Spearman’s rank correlation. *P* < 0.05 was considered the statistical significance.

Supplementary Figure 3 Correlations between plasma ApoM and its-derived indicators with clinical characteristics of AD in subgroup analysis based on sex. ApoM, apolipoprotein M; AD, Alzheimer’s disease; MMSE, mini-mental state examination; MoCA, Montreal cognitive assessment; ADL, activities of daily living; TC, total cholesterol; TG, triglyceride; HDL-C, high-density lipoprotein cholesterol; LDL-C, low-density lipoprotein cholesterol. Correlation analyses were performed using Spearman’s rank correlation. *P* < 0.05 was considered the statistical significance.

Supplementary Figure 4 Correlations between plasma ApoM and its-derived indicators with representative blood biomarkers of AD in subgroup analysis based on APOEε4 status. ApoM, apolipoprotein M; p-tau217, tau phosphorylated at threonine 217; NfL, neurofilament light chain; CN, cognitively normal; AD, Alzheimer’s disease; TC, total cholesterol; TG, triglyceride; HDL-C, high-density lipoprotein cholesterol; LDL-C, low-density lipoprotein cholesterol. Correlation analyses were performed using Spearman’s rank correlation or Pearson’s correlation as appropriate. *P* < 0.05 was considered the statistical significance.

Supplementary Figure 5 Correlations between plasma ApoM and its-derived indicators with representative blood biomarkers of AD in subgroup analysis based on sex. ApoM, apolipoprotein M; p-tau217, tau phosphorylated at threonine 217; NfL, neurofilament light chain; CN, cognitively normal; AD, Alzheimer’s disease; TC, total cholesterol; TG, triglyceride; HDL-C, high-density lipoprotein cholesterol; LDL-C, low-density lipoprotein cholesterol. Correlation analyses were performed using Spearman’s rank correlation or Pearson’s correlation as appropriate. *P* < 0.05 was considered the statistical significance.
