# Supplementary material for: Association Between Plasma Apolipoprotein M With Alzheimer’s Disease: A Cross-Sectional Pilot Study From China
Source: Front Aging Neurosci. 2022 Mar 18;14:838223. doi: 10.3389/fnagi.2022.838223 (PMC8973919; doi:10.3389/fnagi.2022.838223)
Supplement: Supplementary file 1 [file Table_1.docx]

**Supplemental Table 1.** **Pairwise comparison of the area under the receiver operator characteristic curves by DeLong’s test.**

|  | Difference between areas | Z statistic | *p* value |
| --- | --- | --- | --- |
| Model 1 vs. Model 2 | 0.047 | 2.207 | 0.027 |
| Model 1 vs. Model 3 | 0.056 | 2.360 | 0.018 |
| Model 1 vs. Model 4 | 0.067 | 2.847 | 0.004 |
| Model 1 vs. Model 5 | 0.066 | 2.672 | 0.007 |
| Model 1 vs. Model 6 | 0.029 | 1.679 | 0.093 |

Model 1: Age, Sex, *APOE* ε4 positive, BMI.

Model 2: Age, Sex, *APOE* ε4 positive, BMI, ApoM.

Model 3: Age, Sex, *APOE* ε4 positive, BMI, ApoM/TC ratio.

Model 4: Age, Sex, *APOE* ε4 positive, BMI, ApoM/TG ratio.

Model 5: Age, Sex, *APOE* ε4 positive, BMI, ApoM/HDL-C ratio.

Model 6: Age, Sex, *APOE* ε4 positive, BMI, ApoM/LDL-C ratio.

**Abbreviations:** APOE, apolipoprotein E; BMI body mass index; ApoM, apolipoprotein M; TC, total cholesterol; TG, triglyceride; HDL-C, high-density lipoprotein cholesterol; LDL-C, low-density lipoprotein cholesterol. *p* < 0.05 was considered the statistical significance.
