# Supplementary figures and images for: Association Between Plasma Apolipoprotein M With Alzheimer’s Disease: A Cross-Sectional Pilot Study From China
Source: Front Aging Neurosci. 2022 Mar 18;14:838223. doi: 10.3389/fnagi.2022.838223 (PMC8973919; doi:10.3389/fnagi.2022.838223)

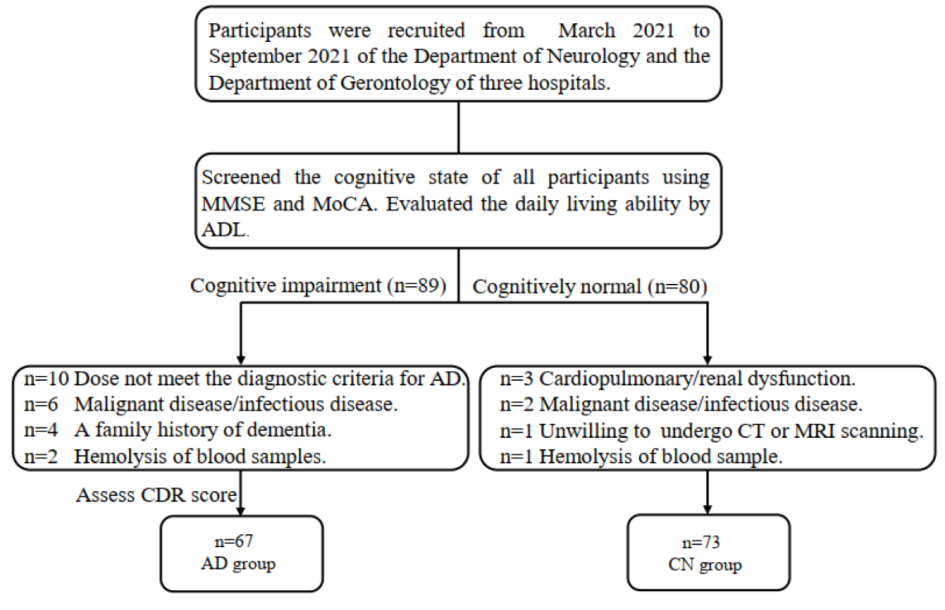

Supplement: Supplementary Figure 1 — The flow diagram for the enrollment of the two groups. MMSE, mini-mental state examination; MoCA, Montreal cognitive assessment; ADL, activities of daily living; CDR-SB, Clinical Dementia Rating; CT, Computed tomography; MRI, Magnetic resonance imaging; AD, Alzheimer’s disease; CN, cognitively normal. [file Image_1.TIFF]

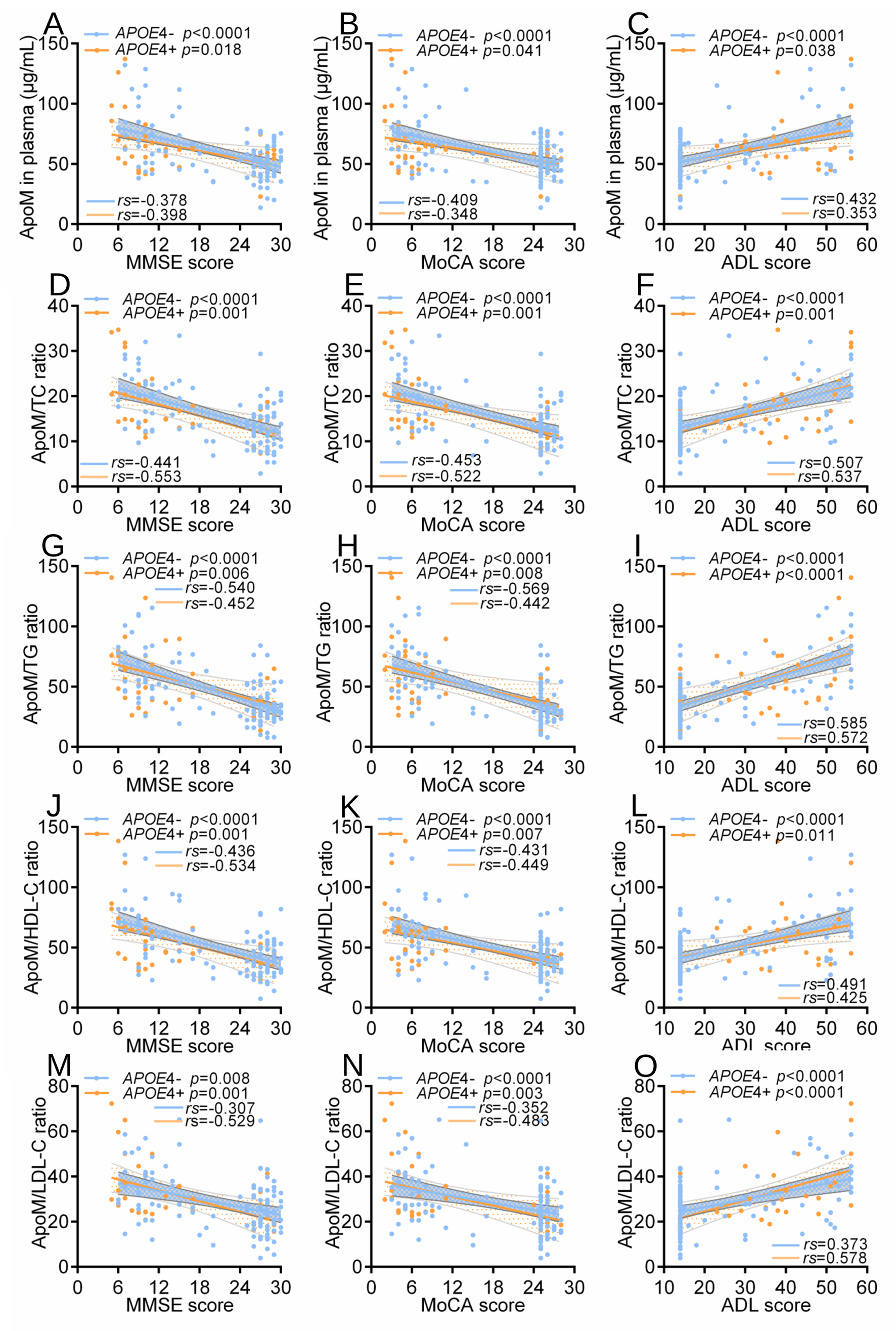

Supplement: Supplementary Figure 2 — Correlations between plasma ApoM and its-derived indicators with clinical characteristics of AD in subgroup analysis based on APOEε4 status. ApoM, apolipoprotein M; AD, Alzheimer’s disease; APOE, apolipoprotein E; MMSE, mini-mental state examination; MoCA, Montreal cognitive assessment; ADL, activities of daily living; TC, total cholesterol; TG, triglyceride; HDL-C, high-density lipoprotein cholesterol; LDL-C, low-density lipoprotein cholesterol. Correlation analyses were performed using Spearman’s rank correlation. P < 0.05 was considered the statistical significance. [file Image_2.TIFF]

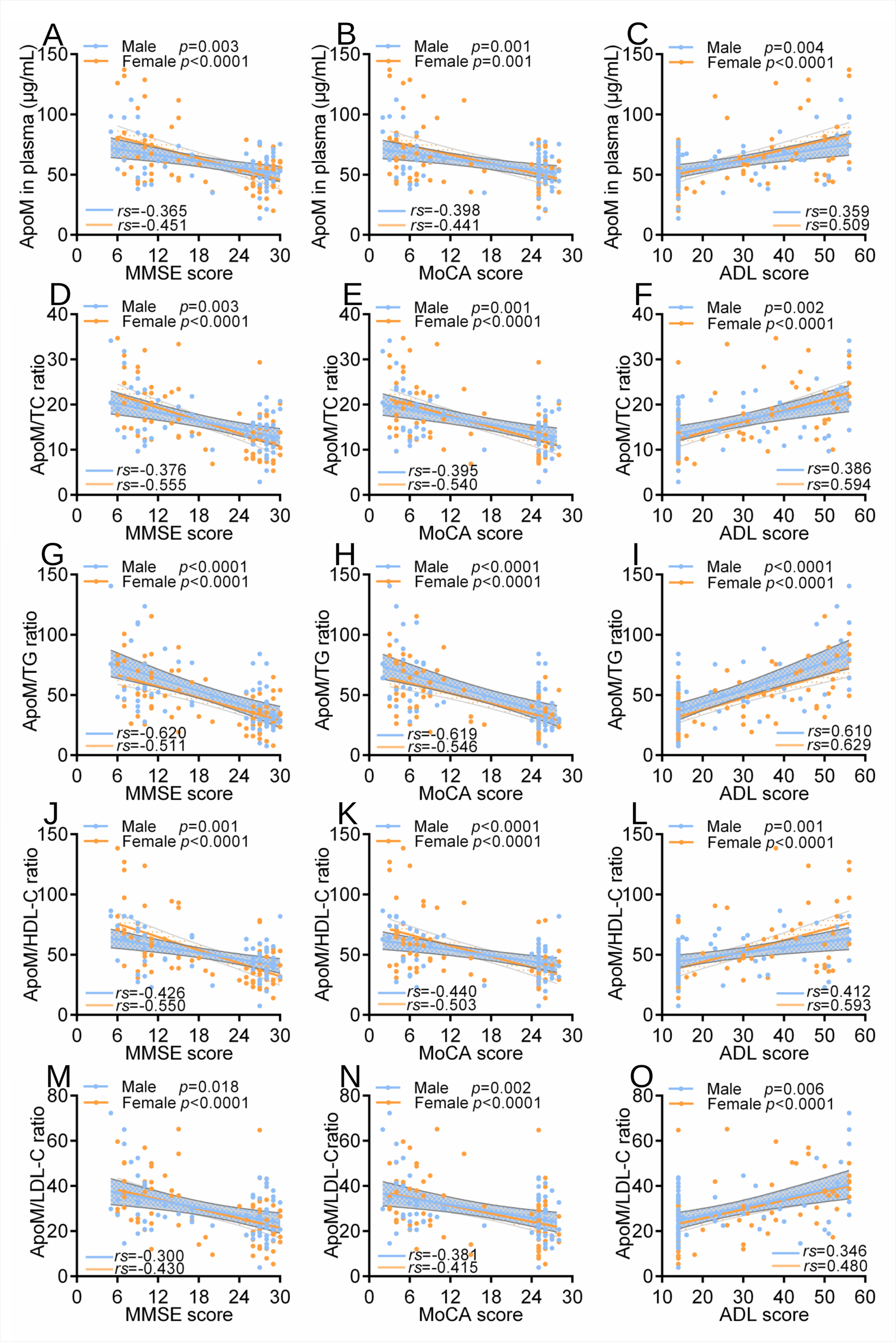

Supplement: Supplementary Figure 3 — Correlations between plasma ApoM and its-derived indicators with clinical characteristics of AD in subgroup analysis based on sex. ApoM, apolipoprotein M; AD, Alzheimer’s disease; MMSE, mini-mental state examination; MoCA, Montreal cognitive assessment; ADL, activities of daily living; TC, total cholesterol; TG, triglyceride; HDL-C, high-density lipoprotein cholesterol; LDL-C, low-density lipoprotein cholesterol. Correlation analyses were performed using Spearman’s rank correlation. P < 0.05 was considered the statistical significance. [file Image_3.TIFF]

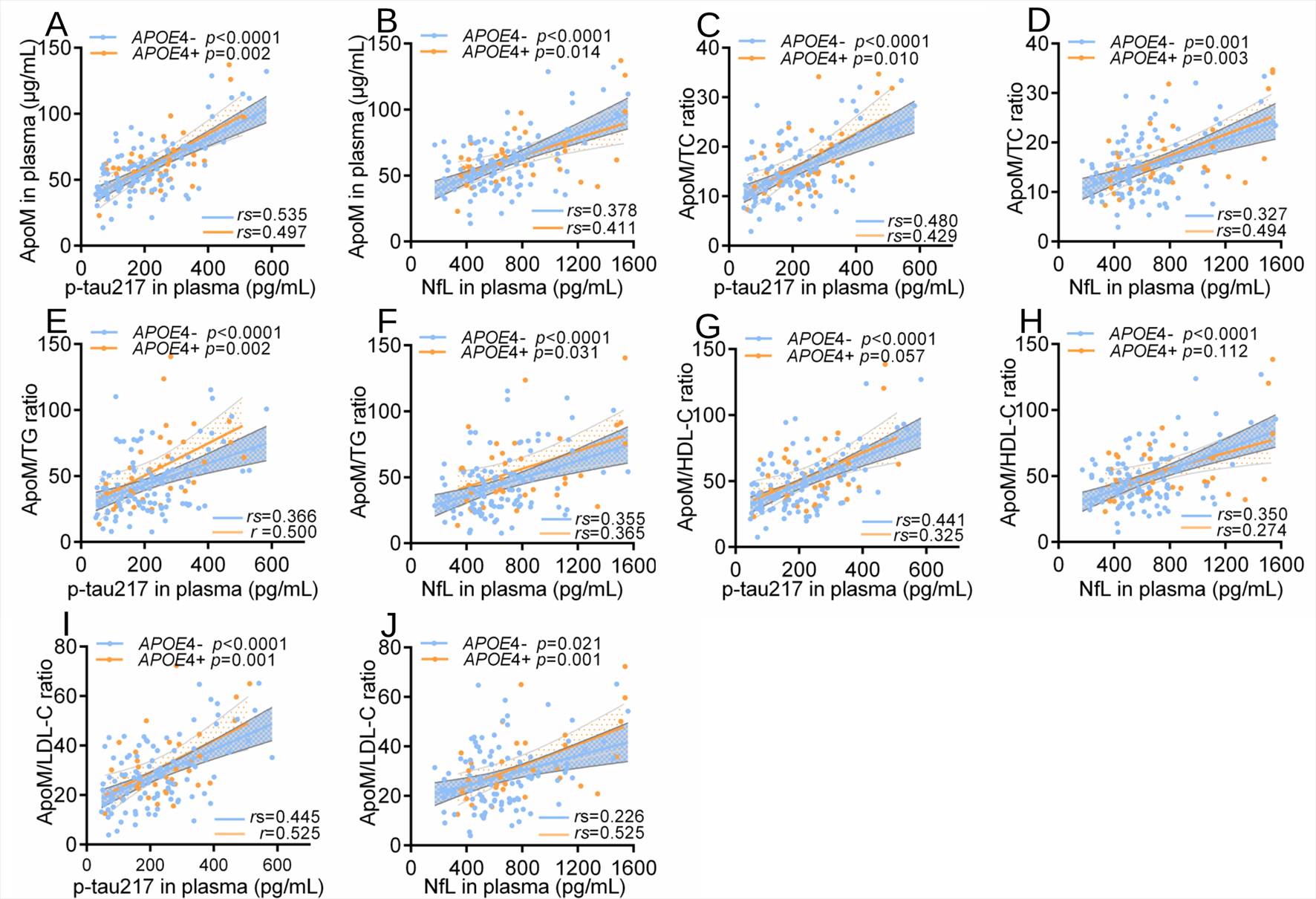

Supplement: Supplementary Figure 4 — Correlations between plasma ApoM and its-derived indicators with representative blood biomarkers of AD in subgroup analysis based on APOEε4 status. ApoM, apolipoprotein M; p-tau217, tau phosphorylated at threonine 217; NfL, neurofilament light chain; CN, cognitively normal; AD, Alzheimer’s disease; TC, total cholesterol; TG, triglyceride; HDL-C, high-density lipoprotein cholesterol; LDL-C, low-density lipoprotein cholesterol. Correlation analyses were performed using Spearman’s rank correlation or Pearson’s correlation as appropriate. P < 0.05 was considered the statistical significance. [file Image_4.TIFF]

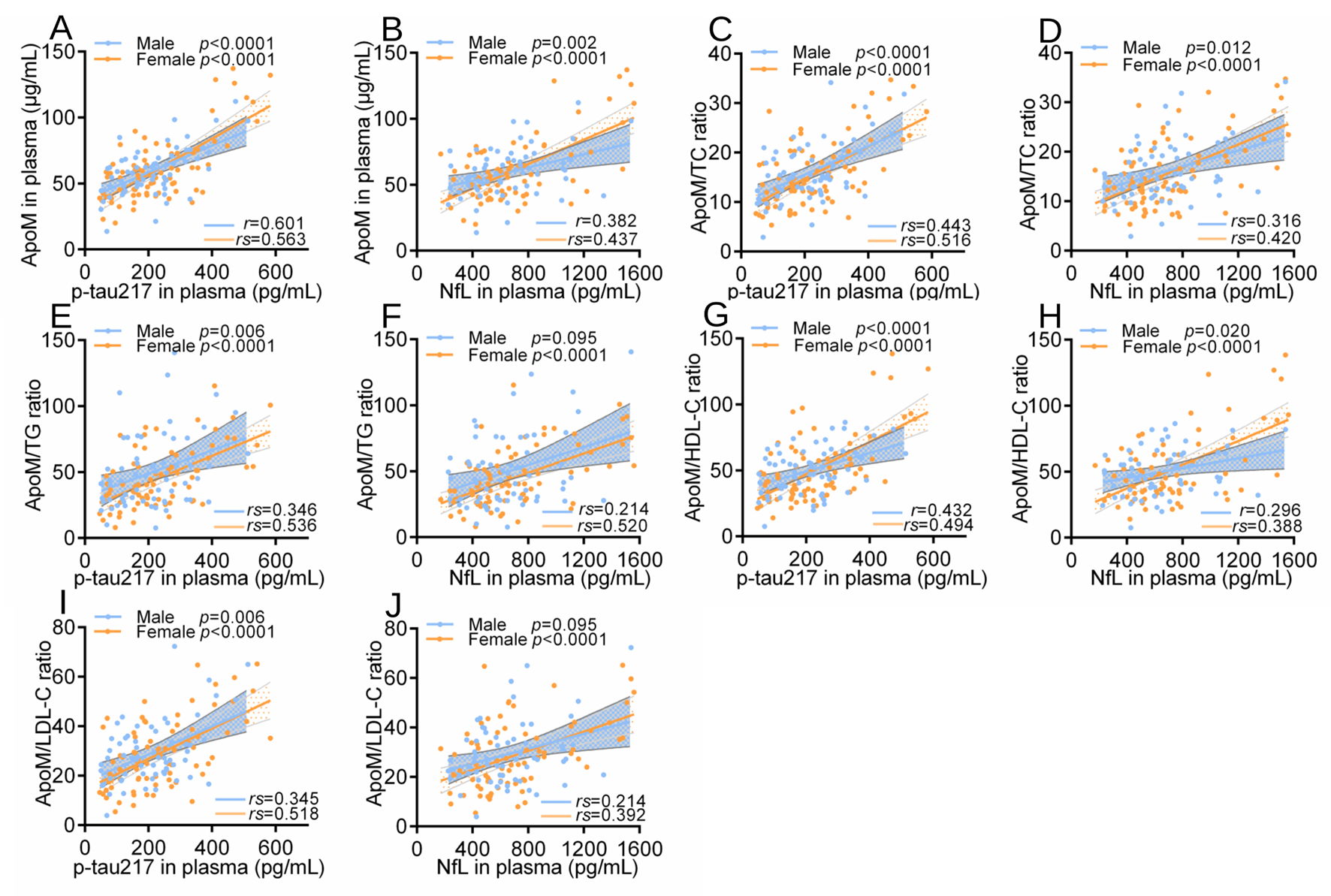

Supplement: Supplementary Figure 5 — Correlations between plasma ApoM and its-derived indicators with representative blood biomarkers of AD in subgroup analysis based on sex. ApoM, apolipoprotein M; p-tau217, tau phosphorylated at threonine 217; NfL, neurofilament light chain; CN, cognitively normal; AD, Alzheimer’s disease; TC, total cholesterol; TG, triglyceride; HDL-C, high-density lipoprotein cholesterol; LDL-C, low-density lipoprotein cholesterol. Correlation analyses were performed using Spearman’s rank correlation or Pearson’s correlation as appropriate. P < 0.05 was considered the statistical significance. [file Image_5.TIFF]
